# G-quadruplex structures trigger RNA phase separation

**DOI:** 10.1093/nar/gkz978

**Published:** 2019-11-13

**Authors:** Yueying Zhang, Minglei Yang, Susan Duncan, Xiaofei Yang, Mahmoud A S Abdelhamid, Lin Huang, Huakun Zhang, Philip N Benfey, Zoë A E Waller, Yiliang Ding

**Affiliations:** 1 Department of Cell and Developmental Biology, John Innes Centre, Norwich Research Park, Norwich NR4 7UH, UK; 2 Organisms and Ecosystems, Earlham Institute, Norwich Research Park, Norwich NR4 7UZ, UK; 3 Department of Biology, Duke University, Durham, NC 27708, USA; 4 School of Pharmacy, University of East Anglia, Norwich Research Park, Norwich NR4 7TJ, UK; 5 Centre for Molecular and Structural Biochemistry, University of East Anglia, Norwich Research Park, Norwich NR4 7TJ, UK; 6 Cancer Research UK Nucleic Acid Structure Research Group, MSI/WTB Complex, The University of Dundee, Dow Street, Dundee DD1 5EH, UK; 7 Key Laboratory of Molecular Epigenetics of Ministry of Education, Northeast Normal University, Changchun 130024, China; 8 Howard Hughes Medical Institute, Duke University, Durham, NC 27708, USA

## Abstract

Liquid–liquid phase separation plays an important role in a variety of cellular processes, including the formation of membrane-less organelles, the cytoskeleton, signalling complexes, and many other biological supramolecular assemblies. Studies on the molecular basis of phase separation in cells have focused on protein-driven phase separation. In contrast, there is limited understanding on how RNA specifically contributes to phase separation. Here, we described a phase-separation-like phenomenon that *SHORT ROOT* (*SHR*) RNA undergoes in cells. We found that an RNA G-quadruplex (GQ) forms in *SHR* mRNA and is capable of triggering RNA phase separation under physiological conditions, suggesting that GQs might be responsible for the formation of the *SHR* phase-separation-like phenomenon *in vivo*. We also found the extent of GQ-triggered-phase-separation increases on exposure to conditions which promote GQ. Furthermore, GQs with more G-quartets and longer loops are more likely to form phase separation. Our studies provide the first evidence that RNA can adopt structural motifs to trigger and/or maintain the specificity of RNA-driven phase separation.

## INTRODUCTION

Liquid–liquid phase separation (LLPS) phenomena, with two liquid phases coexisting in one system, have been recently observed in the cells and shown to be responsible for organization of many complex biochemical reactions temporally and spatially (1). Thus, LLPS is of great importance for many biological processes, including the formation of membrane-less organelles (2,3), the cytoskeleton (4,5), signalling complexes (6,7), and many other biological supramolecular assemblies (8,9).

Previous studies mainly focused on protein-driven phase separation through multivalent interactions. This multivalence can be attributed to at least one of three different specific interactions: (a) proteins with well-defined domains interact with stereospecific interaction surfaces to form oligomers (10); (b) proteins with linker motifs string together to generate linear multivalent interactions (11); (c) proteins featured with intrinsically disordered regions (IDRs) can serve as scaffolds to act with distinctive short linear motifs to form multiple interactions (12–14). Apart from these protein–protein interactions, the specificity for the protein-driven phase separation could also be achieved by the properties of their interacting RNAs including their amount (15), sequence (16) and secondary structure (16,17). For example, the secondary structures of both *CLN3* and *BIN2* RNAs determine the identity of droplets formed by RNA binding protein, whi3, by uncovering or masking its interaction regions (16). Furthermore, ALS/FTD-associated C9ORF72 RNA containing G_4_C_2_ repeat sequences is also involved in the phase separation of G-quadruplex-binding proteins (17).

RNA has generally been considered as a regulatory component in protein-driven phase separation (16). However, there is evidence that RNA itself is also capable of forming phase separation, potentially through multivalent RNA–RNA interactions (18). Examples of these include RNA tandem repeat sequences (19) and RNA homopolymers (20). However, these do not provide any specificity in the formation and/or maintenance of RNA-driven phase separation. Hence, it is still unclear which properties of RNA assure the specificity of RNA-driven phase separation.

In our study, we investigated the properties of *SHR* mRNA, an RNA important in plant development, that was observed to form an RNA phase-separation-like phenomenon *in vivo*. We found that *SHR* contains a G-quadruplex (GQ), a specific RNA tertiary structure motif. G-quadruplexes are composed of G-tetrads formed by Hoogsteen hydrogen bonding and stabilized by π-π stacking interactions and the coordination of cations, especially K^+^ (21). The *SHR-*GQ structure is capable of triggering RNA-driven phase separation. Furthermore, the stability of this GQ, when modified by different conditions, correlated with the extent of phase separation formation. Our study suggests that RNA may adopt specific RNA structure motifs such as G-quadruplexes, to trigger and/or maintain RNA-driven phase separation.

## MATERIALS AND METHODS

### Single molecule FISH (smFISH)

Probes for *SCARECROW* (At3g54220) and *SHORT ROOT* (At4g37650) were designed using the online program Stellaris Probe Designer version 2.0 from LGC Biosearch Technologies (see Supplementary Table S3). smFISH experiments were carried out for *Arabidopsis* roots as previously described (22). Briefly, seedlings were removed from the media and the root tips were cut and fixed in 4% paraformaldehyde for 30 min. The roots were washed twice with nuclease free 1 × PBS (Thermo Fisher Scientific) and then placed onto a poly-l-lysine slide (Thermo Fisher Scientific) and covered by a glass coverslip (R&L Slaughter, Upminster, UK). The meristems were then squashed under the coverslip, before being submerged in liquid nitrogen until frozen. The coverslips were removed using a razor blade and the roots were left on the slide to dry at room temperature for 30 min. Tissue permeabilization was then carried out by immersing the samples in 70% ethanol for a minimum of one hour. Probe hybridization following removal from ethanol, slides were left at room temperature for 5 min before two washes were carried out with wash buffer (10% formamide and 2× saline-sodium citrate buffer; SSC). 100 ml of hybridization solution (10% dextran sulphate, 2× SSC and 10% formamide) containing *SHR*/*SCR* probes (at a final concentration of 250 nM) was added to each slide. Coverslips were placed over the samples to prevent evaporation and the probes were left to hybridize at 37°C overnight in the dark. Excess hybridization solution (containing unbound probes) was pipetted off the following morning. Each sample was washed twice with wash buffer, with the second wash left to incubate for 30 min at 37°C in the dark. After wash buffer removal, 100 ml of the nuclear stain DAPI (4′,6-diamidino-2-phenylindole, 100 ng/ml) was added to each slide and left to incubate at 37°C for 30 min. Following DAPI removal, a 100 ml 2× SSC wash was carried out before 100 ml GLOX buffer (0.4% glucose in 10 mM Tris, 2× SSC) was added to each slide and left to equilibrate at room temperature for 2 min. This was pipetted off and replaced with an anti-fade solution containing 100 ml of GLOX buffer, 1 ml glucose oxidase (Sigma) and 1 ml catalase (Sigma). The samples were then covered by 22 mm × 22 mm No. 1 coverslips (R&L Slaughter, Upminster, UK), sealed with CoverGrip sealant (Biotium, UK) and imaged using a Delta Vision Elite Deconvolution microscope with a ×100 oil immersed objective (NA 1.46).

### Preparation of *in vitro* transcribed RNAs

DNA Templates (see Supplementary Table S3) were amplified and purified, RNAs were *in vitro* transcribed using Hiscribe T7 High Yield RNA Synthesis Kit (New England Biolabs, NEB). Each RNA was purified using pre-casted 6% denaturing polyacrylamide gel (Thermo Fisher Scientific) and the gel band with desired RNA product was cut and soaked in 1× TEL800 buffer (10 mM Tris–HCl pH 7.5, 1 mM EDTA, 800 mM LiCl) overnight, RNA was then purified with ZYMO RNA purification kit (ZYMO S1016).

### Reverse transcription stalling assay

Reverse transcription stalling assay was performed as previously described (23). 500 ng (about 0.3 pmol) of *SHR*-GQ or *SHR*-GQ_m_ RNA was mixed with folding buffer (10 mM Tris–Cl, pH 7.5, 150 mM Li^+^ or K^+^) and denatured at 95°C for 1.5 min. For the reaction with PDS, PDS was added to a final concentration of 5 μM. For reverse transcription, 5× RT buffer (200 mM Tris–HCl, pH 8.3, 750 mM KCl/LiCl, 2.5 mM dNTPs, 12.5 mM MgCl_2_ and 5 mM DTT), 1 μl of 5 μM Cy5-labelled primer and 0.5 μl SuperScript III reverse transcriptase (200 U/μl) (Thermo Fisher Scientific) were added. After reverse transcription, 0.5μl NaOH (1M) was added to the reaction and incubate at 95°C to eliminate RNA template. Finally, 10 μl of 2 × stopping dye solution containing 20 mM Tris–HCl, pH 7.5, 20 mM EDTA, 94% deionized formamide and Orange G was added to the reaction mixture (Orange G dye as tracker). The cDNAs were size fractionated by 8 M urea 8% denaturing polyacrylamide gel. The gel was scanned with typhoon phosphorimager and analyzed by ImageQuant 5.2.

### Circular dichroism (CD)

RNA oligos *SHR*-GQ, *SHR*-GQ_m_, *SHR*-GQ_sc_ (see Supplementary Table S3) were purchased from Gentech Inc. 10 μM RNA in 10 mM Li cacodylate, pH 7.0, was denatured at 95°C for 3 min, cooled to room temperature at the rate of 1°C/min and then incubated at room temperature for 15 min for equilibration. The CD measurements were performed using Applied Photophysics Chirascan-plus with the pathlength of 1 mm, spectra were acquired at each wavelength from 220 to 320 nm at 20°C. Spectrum is an average of four scans with a response time 0.5 s/nm. For data fitting, the K^+^ titration was performed to determine the amount of K^+^ needed for GQ formation. To determine the [K^+^]_1/2_, the CD values at 262 nm as a function of potassium concentration were fit with Origin 8.1 according to the Hill equation.

$$\begin{equation*}\varepsilon \ = \ {\varepsilon _{\rm{F}}} + \frac{{{\varepsilon _{\rm{U}}}{\rm{ - }}{\varepsilon _{\rm{F}}}}}{{{\rm{1 + }}{{\left( {\frac{{\left[ {{{\rm{K}}^ + }} \right]}}{{\left[ {{\rm{K}}_{1/2}^ + } \right]}}} \right)}^{{n}}}}}\end{equation*}$$

where ϵ_F_ is the normalized ellipticity of fully folded CD signal, and ϵ_u_ is the normalized ellipticity of unfolded CD signal, [K^+^]_1/2_ is the concentration of potassium ion needed for 50% of the RNA to fold into GQ, and n is the Hill coefficient.

### RNA structure prediction

To predict the RNA secondary/tertiary structure, *SHR* sequence was subjected to *ViennaRNA* web server (24), with adjustment of temperature to 22°C to match the growth temperature of *Arabidopsis*. For the GQ prediction, *GQRS mapper* (25) prediction was used with the default settings.

### Phase separation assay

RNAs and K^+^ was mixed with concentration shown on the phase diagram (Figure 6). Before the experiment, RNA, K^+^ and 10% PEG_8000_ were mixed thoroughly, the mixture was then heated to 95°C for 3 min and cooled down at 0.5°C/min to room temperature and imaged immediately. RNA was stained by nucleic acid dye SYTO 17 (Sigma, S7579). Samples were visualized by a confocal microscope (ZEISS LSM 880) with ×40 oil immersion objective.

### Quantification of droplets in ROI (region of interest)

The extent of phase separation was quantified by the index of dispersion (σ^2^/μ) of fluorescence intensity per pixel (pixel size 43 nm × 43 nm). In brief, for each ROI, the variance of fluorescence intensity was determined, and normalized by the mean RNA fluorescence intensity in the solution phase. 10 independent imaging areas (∼2500 μm^2^ each) were analyzed for each condition.

## RESULTS AND DISCUSSION

### *SHR* mRNA displays a phase separation like phenomenon in cells

**Figure 1.**
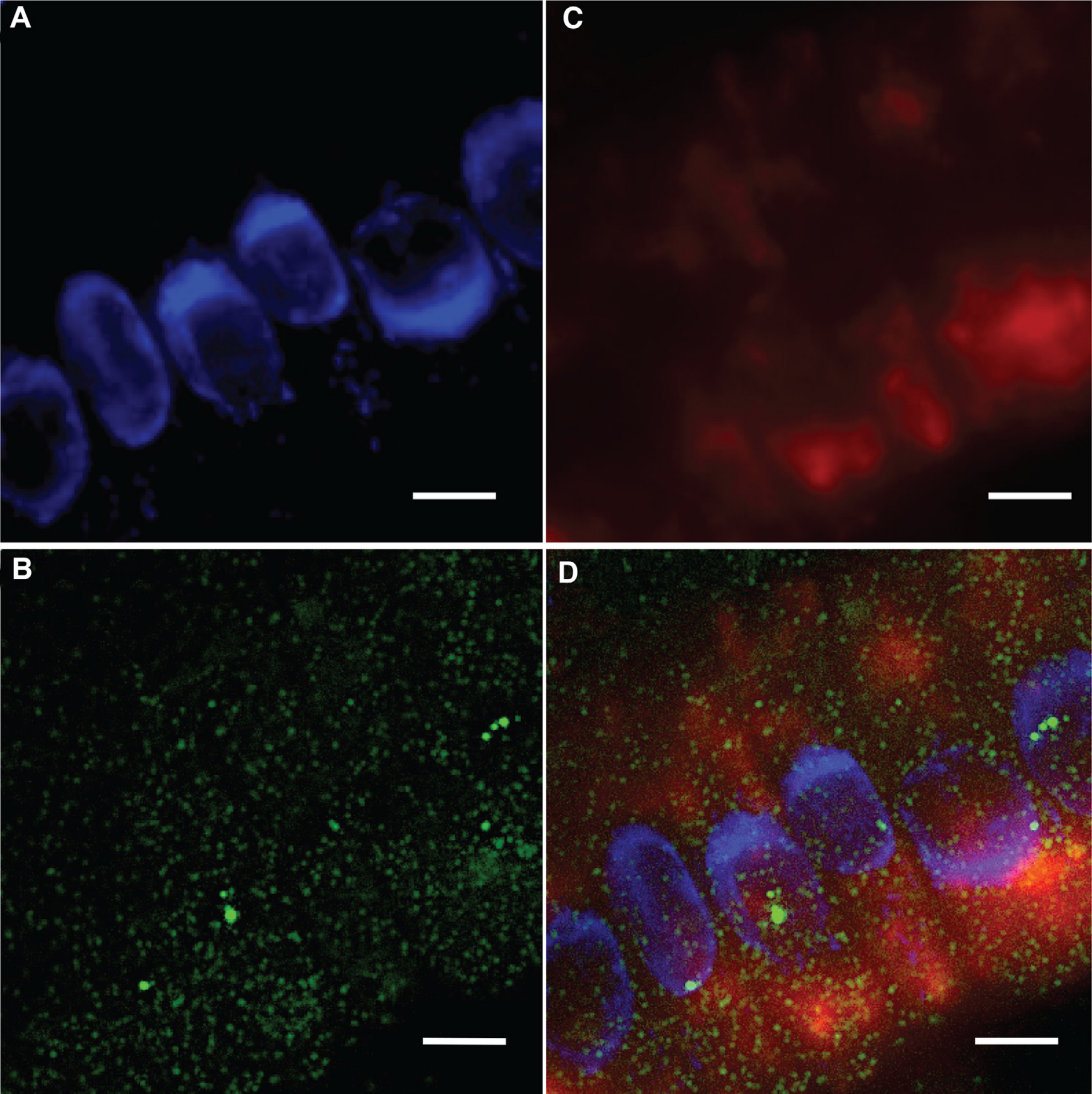
Phase-separation-like *SHR* and punctate *SCR* RNA labelling observed in plant root cells, images generated by smFISH. (**A**) Nuclei stained with DAPI (blue), *SCR* RNA (green) is shown in (**B**) and *SHR* RNA (red) in (**C**). Merged image in (**D**). Scale bar, 10 μm.

Initially our work focused on visualizing the mRNA distribution of *SHORT ROOT* (*SHR*) and *SCARECROW* (*SCR*), two key genes for *Arabidopsis* root development (26,27), using single molecule fluorescence *in situ* hybridization (smFISH). This is a technique which utilizes fluorescence-labelled antisense-probes to visualize individual RNA molecules *in vivo* (28). We used DAPI (4′,6-diamidino-2-phenylindole) to stain the nuclei (Figure 1A, Supplementary Movie S1). We found that the signals for *SCR* RNA were evenly distributed in cells, with each cytosolic green spot of a uniform signal intensity representing a single *SCR* RNA molecule (Figure 1B, Supplementary Movie S1). This even distribution of RNAs is typical for mRNA localization (29,30). Surprisingly, in the same cells, we observed aggregated, phase separation-like signals for *SHR* RNAs (Figure 1C, Supplementary Movie S1). After merging the nuclei and RNA images, we determined that *SHR* signals were mainly localized to the cytoplasm (Figure 1D, Supplementary Movie S1).

To further investigate this phase-separation-like phenomena in cells, we performed another smFISH experiment on a transgenic line with inducible *SHR* (*shr-2 pSHR::SHR:GR*), where the expression of *SHR* was induced by dexamethasone(2). Following the dexamethasone induction, we observed that the big globular-shape signals of *SHR* RNA appeared in cells after 2 h (Supplementary Figure S1, Supplementary Movie S2). These big globular-shaped signals were very different from the dot-shaped signals (such as *SCR*), indicating a phase separation-like phenomenon and this phase separation-like phenomenon is specifically associated with *SHR* expression.

Our smFISH results indicate that *SHR* mRNA displays a phase-separation-like phenomenon in plant cells. As a critical gene in ground tissue patterning and identity maintenance, *SHR* plays important roles in root development (26,31,32). During root pattern formation, SHR proteins are translated in the stele and move from the stele into the endodermis where they interact with SCR. This interaction leads to restriction of SHR movement to a single cell layer of endodermis (26,31,32). While the focus to date has been on SHR protein movement and activity, the cellular concentration and localization of *SHR* mRNA may also affect *SHR* function. The phase-separation-like phenomenon of *SHR* we observed might result in the sequestering of *SHR* mRNA enhancing or suppressing its translational efficacy. Thus, our observations lead to a new hypothesis that plant root cells might organize the cellular *SHR* mRNA levels in space and time to assure the accurate regulation of root development.

### A G-quadruplex forms in *SHR* mRNA

**Figure 2.**
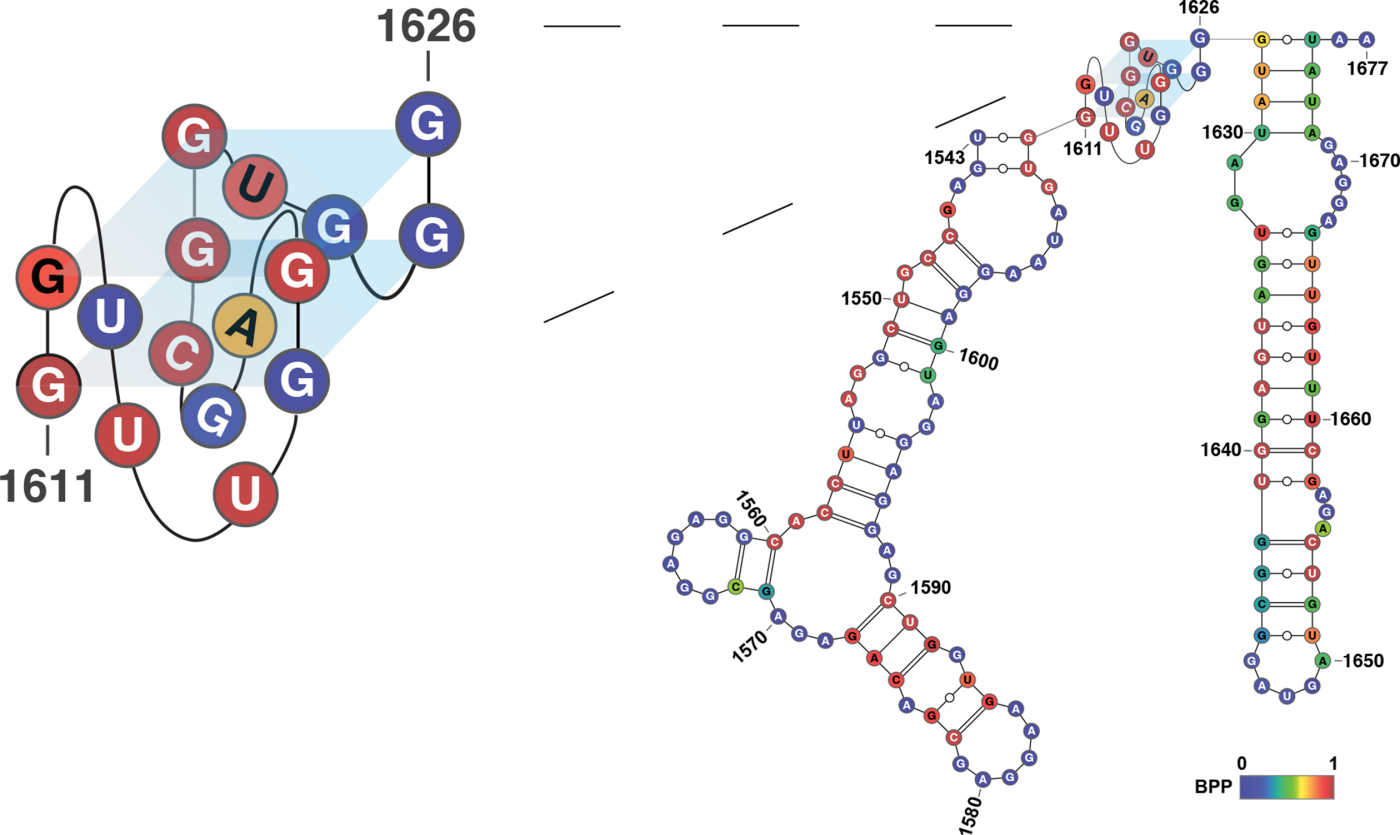
Formation of GQ in *SHR* mRNA. RNA structure model of *SHR*, showing the fragment with GQ structure, predicted by *ViennaRNA* (24). A parallel topology is shown (supported by circular dichroism studies), nucleotides are color-coded according to base-pairing probability (BPP), where 1 represent 100% double-stranded, 0 represent 100% single-stranded. GQ structure is enlarged for better visualization.

To identify the molecular properties of *SHR* RNAs capable of producing this phase-separation-like phenomenon, we investigated both *SHR* mRNA sequence and secondary/tertiary structures. Previous studies showed that multivalent RNA–RNA interactions through RNA tandem repeat sequences are able to form phase separation (19). Therefore, we searched the *SHR* sequence using *tandem repeat finder* (33) but found no tandem repeats, suggesting RNA phase separation was caused by an alternative trigger. Single stranded regions of RNA have also been implicated as the basis for multivalent interactions (16). Additionally, stable secondary structures might inhibit the phase separation by masking the flanking single-strandedness, which could contribute to multivalent interactions (16). Using *ViennaRNA* (24) to predict secondary/tertiary structures formed in *SHR* RNA, we found a quite stable RNA structure without any long single-stranded loop regions or predicted pseudoknot structures (Figure 2, Supplementary Table S1).

Interestingly, we found a putative G-quadruplex (GQ) motif, was predicted at position 1611–1626 (Figure 2). An RNA G-quadruplex is one specific tertiary structure motif that could form in a special sequence context of G_*x*_L_*a*_G_*x*_L_*b*_G_*x*_L_*c*_G_*x*_ (G stands for guanine; L stands for loop), where *x* ≥2, and *a*, *b*, *c* are ≥1 nt and ≤7 (34). This putative GQ motif was also predicted using *QGRS mapper*, a program designed to predict GQ forming sequences (25). We found that the GQ motif predicted by *ViennaRNA* had the highest confidence score among all the putative GQs found by *QGRS mapper* in the sequence (Supplementary Table S2). This putative GQ motif predicted in *SHR* is a two-guanine-quartet (G2) GQ, with the sequence motif as G_2_L_3_G_2_L_3_G_2_L_2_G_2_ (Figure 2).

**Figure 3.**
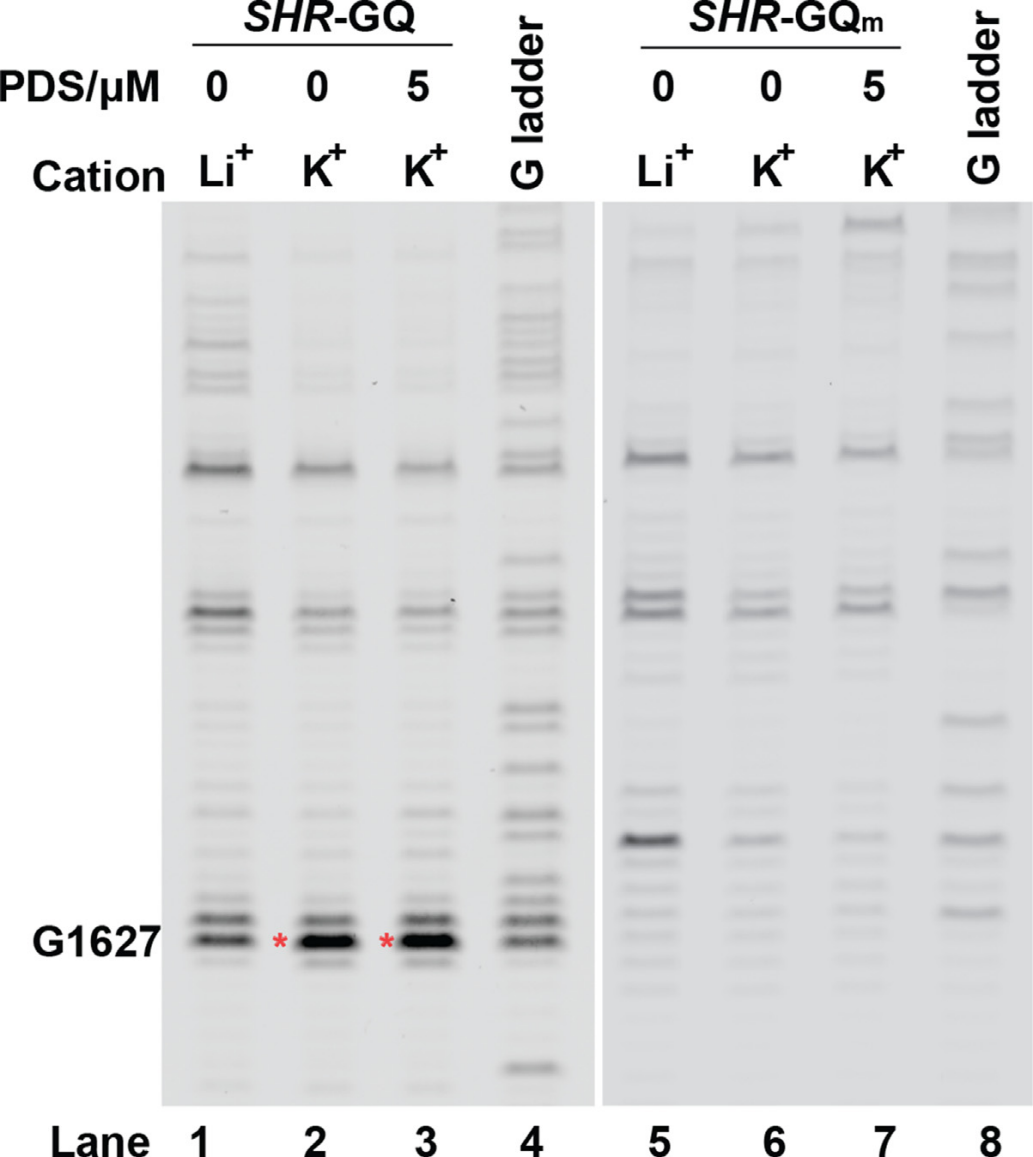
Reverse transcription stalling assays of *SHR*-GQ RNA shows K^+^ dependent stalling (red asterisk) at one nucleotide after the folded GQ (lane 2 and lane 3), *SHR*-GQ_m_ do not have stalling at the corresponding position (lane 6 and lane 7). G-ladders (lane 4 and lane 8) show the sequencing lanes of G for *SHR*-GQ (lane 4) and *SHR*-GQ_m_ (lane 8), respectively.

To probe the presence of this GQ in *SHR* mRNA, we performed a reverse transcription (RT) stalling assay (35). The GQ formation is typically stabilized by potassium (K^+^) but not lithium (Li^+^), and GQs may be preferentially stabilized by using both potassium and the G-quadruplex stabilizing ligand pyridostatin (K^+^+PDS) (36). If GQ is stably formed, the reverse transcriptase will stall during reverse transcription at positions where there is a folded RNA G-quadruplex (23). We observed stronger RT stalling signals in both K^+^ and K^+^+PDS as compared to Li^+^ conditions (lanes 1–3, Figure 3), confirming the formation of this GQ in *SHR* mRNA under the K^+^ and K^+^ + PDS conditions.

To further confirm that the stalling was caused by GQ folding, we used a mutated *SHR*-GQ_m_ RNA, in which the Gs that contribute to G-quartets were mutated to As (Supplementary Table S3). No RT stalling was observed for the *SHR*-GQ_m_ RNA (lanes 5–7, Figure 3), providing further evidence that RT stalling is due to GQ formation in *SHR*.

### *SHR* G-quadruplex forms under physiological conditions

**Figure 4.**
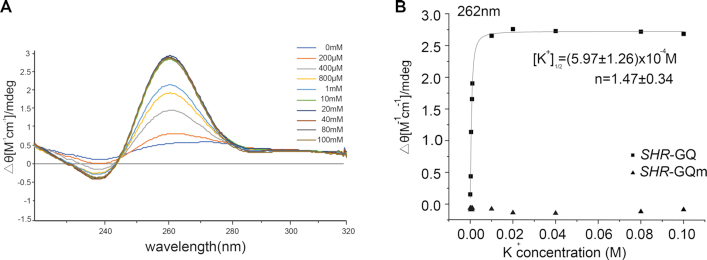
*SHR*-GQ forms a parallel GQ structure. (**A**) Circular Dichroism profile of *SHR*-GQ RNA with potassium ion titration. (**B**) Circular Dichroism signal (ellipticity monitored at 262 nm) of *SHR*-GQ (squares) and *SHR*-GQ_m_ (triangles) are shown as a function of K^+^ concentration. Data were fitted with Hill equation. The [K^+^]_1/2_ and Hill coefficients (*n*) are provided in the plot.

To determine the type of GQ formation in *SHR* we used circular dichroism (CD) (37). A titration with K^+^ was performed and we observed a spectrum with a positive signal at 262 nm and a negative signal at 242 nm, indicative of a parallel-type GQ structure (Figure 4A) (38,39). The K^+^ titration CD profile fitted well to a two-state model with a [K^+^]_1/2_ value of (5.96 ± 1.26) × 10^−4^ M (Figure 4B).

The formation of GQ reached saturation at ∼10 mM, indicating strong GQ formation (Figure 4B). In contrast, the mutated *SHR*-GQ_m_ RNA did not show any GQ spectrum signature at any concentration of K^+^, only giving a spectrum consistent with a random coil, suggesting a complete abolition of GQ formation (Figure 4B, Supplementary Figure S2A). These CD results combined with the RT stalling experiments confirmed the formation of a parallel-type GQ structure in *SHR* mRNA. Notably, this GQ could be triggered and stabilized at very low potassium level ([K^+^]_1/2_ is ∼0.6 mM) (Figure 4B). Since the physiological K^+^ concentration is ∼100 mM (40), it is most likely that this GQ forms and is stable in plant cells.

### *SHR* G-quadruplex can trigger phase separation

**Figure 5.**
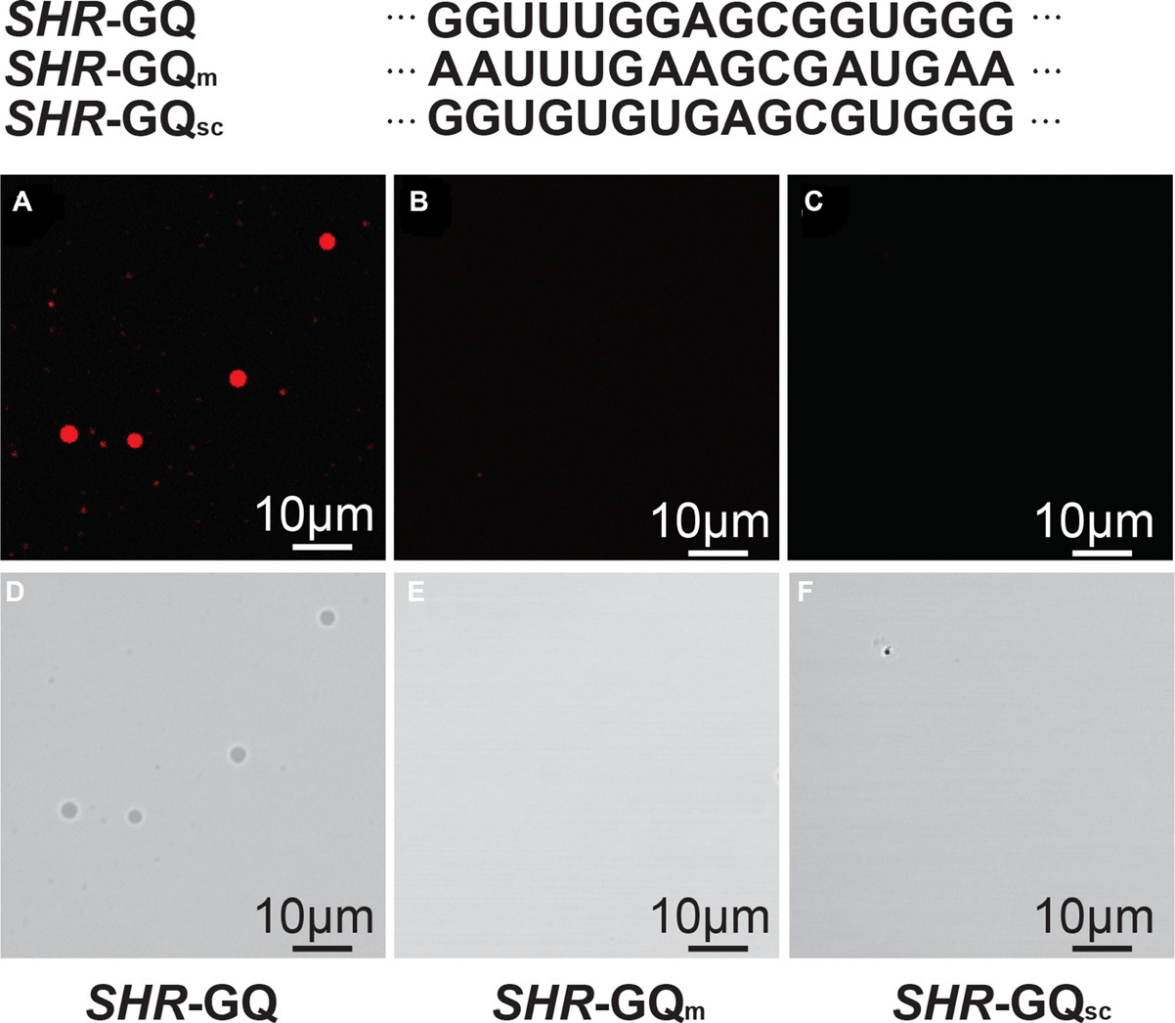
GQ is able to trigger the liquid–liquid phase separation. Fluorescence micrographs of *SHR*-GQ (**A**, **D**), GQ-mutation (*SHR*-GQ_m_) (**B**, **E**) and GQ-scramble (*SHR*-GQ_sc_) (**C**, **F**) RNAs at the physiological K^+^ concentration. Scale bars = 10 μm

We next asked if this GQ motif is able to trigger phase separation of *SHR* mRNA. We performed a phase separation assay on *SHR*-GQ and *SHR*-GQ_m_ RNAs at the physiological K^+^ concentration (100 mM K^+^) (40) and found that *SHR*-GQ formed clear droplets (Figure 5A, D) whereas the mutant (*SHR*-GQ_m_) was not able to trigger phase separation (Figure 5B, E).

**Figure 6.**
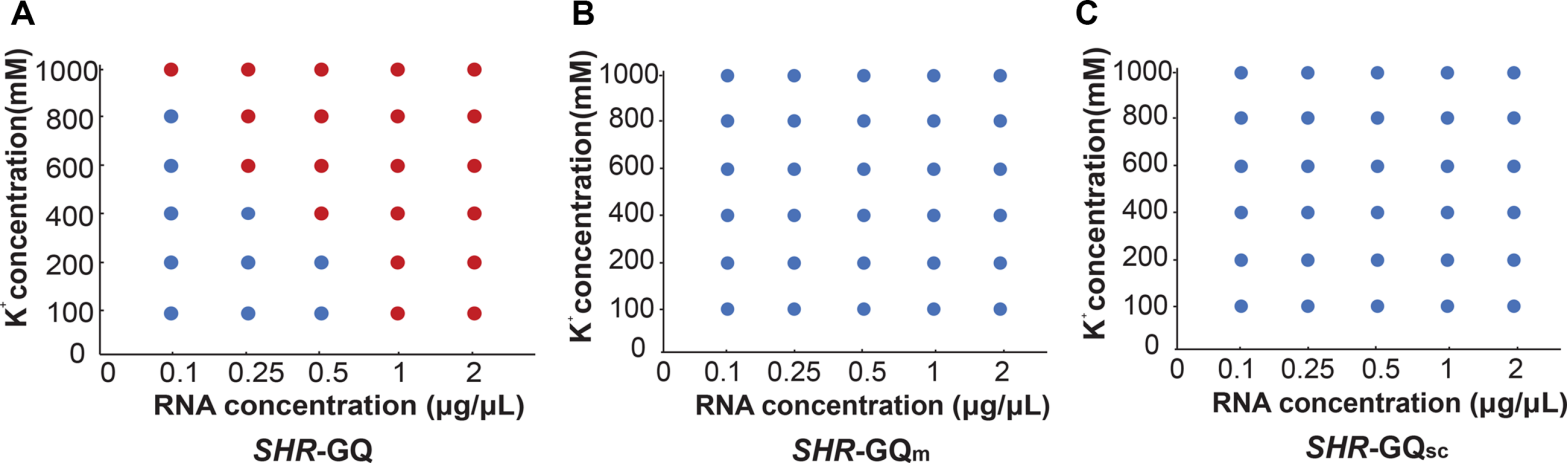
Phase diagrams of *SHR*-GQ (**A**), *SHR*-GQ_m_ (**B**) and *SHR*-GQ_sc_ (**C**) under different RNA and K^+^ concentrations. Red dots indicate where liquid–liquid-phase-separation occurs; blue dots indicate a lack of liquid–liquid-phase-separation.

This result provided evidence that this *SHR*-GQ is capable of triggering the formation of phase separation (Figure 5A, D). We further generated another mutated GQ (*SHR*-GQ_sc_), in which the GQ sequence was scrambled to avoid intramolecular GQ formation (Supplementary Table S3). As expected, *SHR*-GQ_sc_ did not show the same responsiveness to K^+^ (Supplementary Figure S2B), producing only very weak GQ signals at high K^+^ concentration. This suggests that *SHR*-GQ_sc_ is not able to form intramolecular GQ but might be able to form weak GQ through intermolecular interactions (Supplementary Figure S2B). The phase separation assay for *SHR*-GQ_sc_ showed *SHR*-GQ_sc_ that was unable to trigger phase separation (Figure 5C, F). In the following systematic assessment of the capability of *SHR*-GQ to form phase separation under different K^+^ and RNA concentrations, we found that the extent of phase separation triggered by *SHR*-GQ increased with the rise of RNA concentration as well as with K^+^ concentration (Figure 6A, Supplementary Figure S3).

**Figure 7.**
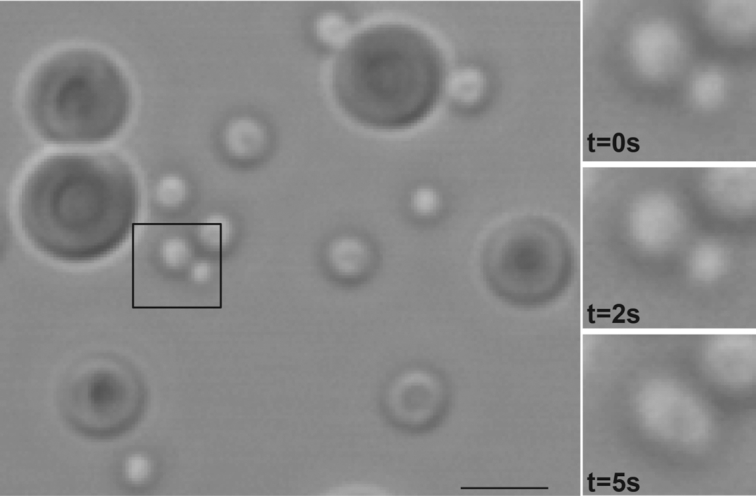
The quick rearrangement of GQ-triggered droplets. Left, droplets formed by *SHR*-GQ. Right, droplets formed by *SHR*-GQ undergo fusion over time. Scale bar = 5 μm.

In contrast, neither the *SHR*-GQ_m_ nor *SHR*-GQ_sc_ mutants were able to trigger phase separation, even at high K^+^ concentration (1000 mM) and RNA concentration (2 μg/μl) (Figure 6B, C). These results indicate that the specificity of RNA-driven phase separation is provided by the *SHR* intramolecular GQ structure. We next investigated the physical properties of the *SHR*-GQ triggered droplets, these droplets were found to undergo rapid rearrangement and fusion events (Figure 7, Supplementary Movie S3), indicating that the droplets were in a relaxed, liquid-like state.

To further determine whether GQ structure act as a general trigger of phase separation when embedded within any RNA sequence, we generated another two mutated sequences (*SHRscramble1-*GQ and *SHRscramble2-*GQ), in which the flanking sequences of GQ were scrambled (Supplementary Table S3). We found that both RNAs were able to trigger RNA phase separation (Supplementary Figures S4 and S5), suggesting that GQ serves as a general trigger of RNA phase separation. To further confirm the full-length *SHR* is capable of triggering RNA phase separation at the physiological K^+^ concentration, we performed the *in vitro* phase separation assay on the RNAs with full-length *SHR* and full-length *SHR* GQ mutants (Supplementary Table S3). We found that the full-length *SHR*, similar to the short-length *SHR*, was able to trigger RNA phase separation at physiological K^+^ concentration (Supplementary Figure S6), while the full-length *SHR* GQ mutant was not capable of triggering RNA phase separation (Supplementary Figure S6). By increasing the potassium concentration up to 400 mM, we observed RNA phase separation of the full-length *SHR* GQ mutant (Supplementary Figure S7), which might be due to non-specific helical stacking (18). Overall, the full-length *SHR* is capable of triggering RNA phase separation at physiological K^+^ concentration, further confirming that *SHR* is likely to trigger RNA phase separation in cells.

Taken together, our results indicate that *SHR*-GQ is capable of triggering liquid–liquid phase separation through intramolecular GQ structure formation. Notably, this RNA G4 located within *SHR* coding region. It is possible that *SHR*-GQ driven RNA phase separation might act as a regulator/factor for *SHR* translation.

### Properties of GQ affect the extent of GQ-triggered phase separation

**Figure 8.**
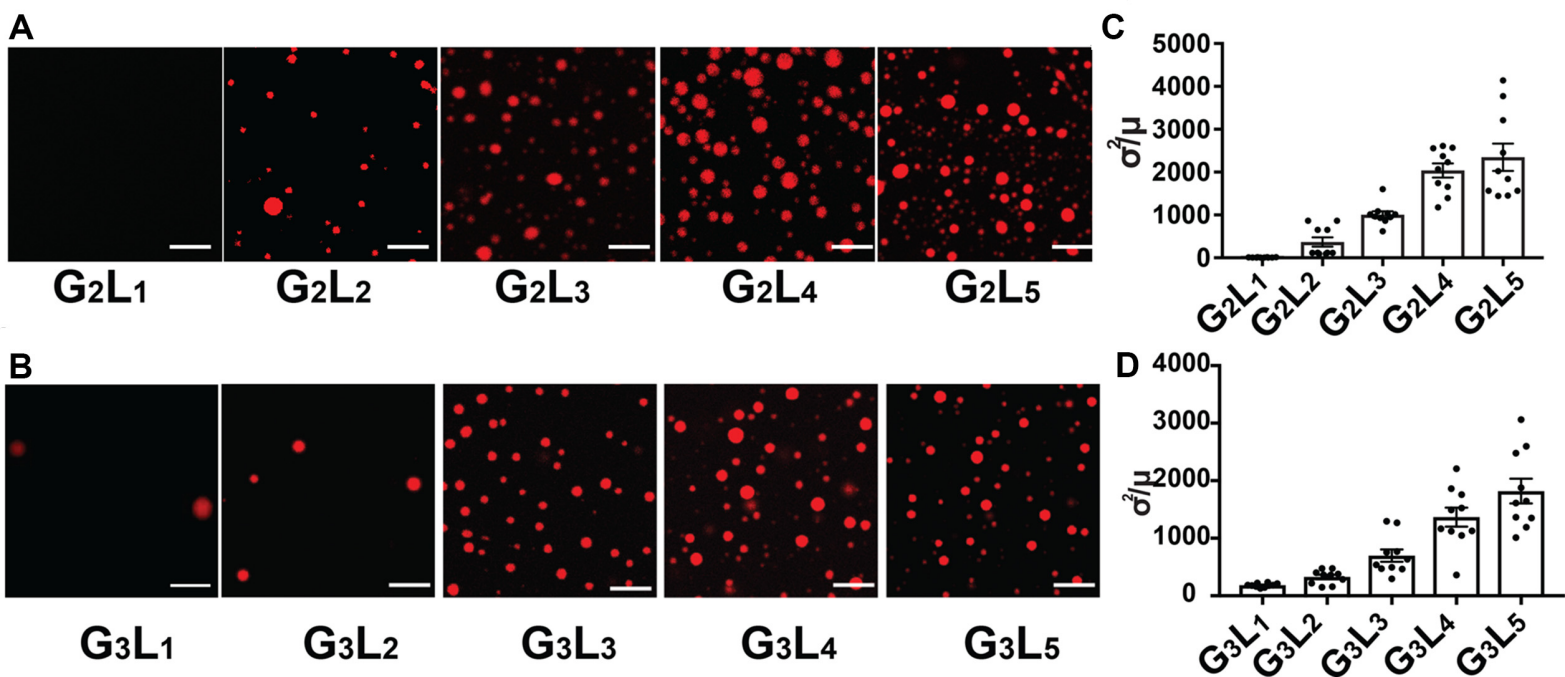
The capability for GQ-triggered-phase-separation is affected by the number of G-quartets and loop lengths. (**A**) Fluorescence micrographs of phase separation of G2 GQ RNAs with different loop lengths (1 μg/μl RNA under 800mM K^+^). (**B**) Fluorescence micrographs of phase separation of G3 GQ RNAs with different loop lengths (1 μg/μl RNA under 400 mM K^+^). (**C**) Quantitation of inhomogeneity as normalized variance (σ^2^/μ) of phase separation for G2 GQ RNAs with different loop lengths. (**D**) Quantitation of inhomogeneity as normalized variance (σ2/μ) of phase separation for G3 GQ RNAs with different loop lengths. Data are shown as mean ± SE.

Previous studies indicate that the stability of GQ forming sequences is generally determined by the number of G-quartets and the length of the loops (39,41). To probe the relationship between phase separation and stability of the GQ forming sequence, we determined the capacity for phase separation of sequences with different numbers of quartets (G = 2 or 3) and different loop lengths (L = 1 to 5) (see Supplementary Table S3 for full list). We found that the G3 GQs tend to trigger phase separation under lower K^+^ concentrations and lower RNA concentrations as compared to G2 GQs (Figure 8A, B, Supplementary Figures S8 and S9).

Our results indicate that the G3 GQs provide a better trigger for phase separation as compared to G2 GQs and provides further evidence that the GQ structure is responsible for the phase separation formation. Interestingly, we also found that the GQs with longer loop lengths tend to form more droplets as compared to those with shorter loop lengths (Figure 8A, B, Supplementary Figures S8 and S9). The index of dispersal (σ^2^/μ) in Region Of Interest (ROI) was used to quantify the extent of phase separation (19). Consistently, longer loop lengths resulted in an increased extent of droplets formation (Figure 8C, D). The longer loops might allow more multivalent interactions among GQs. Overall, our results indicate that GQs with more G-quartets and longer loops have a greater extent of triggering phase separation. From a previous prediction of GQs across *Arabidopsis* genome, G2s with longer loops (G_2_L_3–4_) are shown to be more dominant among all the GQ types (42). Given that in our experiments G2 with longer loops (L = 3 to 5) triggered phase separation more easily (Supplementary Figure S8), this suggested that these G2 longer-loop GQs in *Arabidopsis* are likely to form similar RNA-driven phase separation as *SHR*-GQ under physiological conditions.

RNA GQs have been suggested to influence translation in the plant development (34,43). In plants, the cellular levels of K^+^ can vary between different developmental stages and stress conditions (44,45). For instance, under drought conditions, plants increase cytosolic K^+^ concentrations from 100 mM under normal cellular conditions up to 700 mM in order to avoid cellular dehydration (46). This dramatic increase of K^+^ concentration in the cell is likely to promote more GQ to form strongly, subsequently inducing more GQ-triggered RNA-driven phase separation. This GQ-triggered RNA-driven phase separation might be a regulatory mechanism for the cells to protect and/or store RNAs under the stress conditions such as drought.

In conclusion, our results provide the first demonstration of RNA adopting a specific RNA structure, e.g. GQ, to allow the formation of RNA-driven phase separation. This RNA-structure-triggered phase separation might be responsible for our observation of the phase-separation-like phenomenon of *SHR in vivo*, providing the possibility of a new regulatory role in plant root development. It also reveals a new functional role for GQ structures, serving as a trigger for RNA-driven phase separation.

## Supplementary Material

gkz978_Supplemental_Files
