# Supplementary material for: G-quadruplex structures trigger RNA phase separation
Source: Nucleic Acids Res. 2019 Nov 13;47(22):11746–54. doi: 10.1093/nar/gkz978 (PMC7145655; doi:10.1093/nar/gkz978)
Supplement: gkz978_Supplemental_Files [file gkz978_supplemental_files.zip › Revised_Supplementary_Information-.docx]

**G-quadruplex Structures Trigger RNA Phase Separation**

Yueying Zhang,^1‡^ Minglei Yang,^1‡^ Susan Duncan,^1,2,6‡^ Xiaofei Yang,^1^ Mahmoud A. S. Abdelhamid,^3,4^ Lin Huang,^5^ Huakun Zhang,^1,7^ Philip N. Benfey,^6,8*^ Zoë A. E. Waller,^3,4*^ Yiliang Ding^1*^

**Supplementary Information**

**Table S1. The minimum free energy (MFE) secondary structure of *SHR* mRNA predicted by *ViennaRNA* in dot-bracket notation.**

**Table S2. *GQRS mapper* prediction results.**

**Table S3. DNA/RNA sequences used in this study.**

**Figure S1. *SHR* RNA foci in the root cells of the inducing *SHR* transgenic lines in *shr-2* mutant**

**Figure S2. Circular Dichroism profiles of RNAs.**

**Figure S3. Phase separation of *SHR*-GQ under different conditions.**

**Figure S4. Droplets formed by *SHR-*GQ , *SHRscramble1-*GQ and *SHRscramble2-*GQ.**

**Figure S5.** **GQ as a general trigger for phase separation.**

**Figure S6. Full-length *SHR* RNA formed phase separation under physiological conditions.**

**Figure S7. Phase diagram of Full-length *SHR* RNA and Full-length *SHR* RNA with GQ mutation.**

**Figure S8. Phase diagram of G2-GQ Forming Sequences.**

**Figure S9. Phase diagram of G3-GQ Forming Sequences.**

**Movie S1. Z sections of plant roots with smFISH staining of *SHR* and *SCR* RNAs.**

**Movie S2. Z sections of plant roots with smFISH staining of *SHR* in the *SHR* inducing transgenic line.**

**Movie S3. The quick rearrangement of GQ-triggered droplets**

**References**

**Table S1. The minimum free energy (MFE) secondary structure of *SHR* mRNA predicted by *ViennaRNA* in dot-bracket notation**

1 .......(((((....(((((((........(((.....))).((((..((((......((((((((((.(((((((.((((.....((((((((((((.....((((........))))...)))..)).)))))))((....))..)))).)))..))

160 ))...)))((((((.......(((((((((.........(((((....)))))(((((((.(((((.(((((((..((((..............(((....)))..(((((((((((.(((....))).((....((((((((((((((...((....((

320 ........)).(((((.((((....))))((((((((((.....((((((((.......)))))))).(((((......((((((((((((.........((........))........(((((((...........(((((((((((((..((((.((

480 ((((((.......((((((((.((((((((((((((((((((((((((((..(((.........((((((.((((((((((((..((((.(((((.((.(((....))).((..(((((..((((.((((((.......)))).((((((.(((((((((

640 ((..(((.......)))....))))))))........(((...)))))))))).))((((...(((((....)))))....))))....))))))(((((.((((.(((.((((((((((.((((((((((((((((.(((.......)))...))))).

800 .((.(((.(((((..(((......(((.(((((((((((........)))).((((.......)))).......(((.(((.....(((((((......)))))))))).))))))))))))))))..))))).(((((((((((((((((....)))((

960 .((.......)).))..))))).)))..(((((((.((((......((....))...))))))..(((((..((((......)))).(((....))))))))))))).....))))))..((((...(((((((((((((.(((((((((((.((((((.

1120 ..))))..(((....))).....((((....)))))))))))).............)))))..((((((......)))(((((((.......))))))))))(((((.((((((...))).)))...))))).(((....)))))))).((((...((((

1280 (.....)))))))))..))))))))...)))).))).))..((((....)))).)).))))).))))....)))).)).))))(((...)))))))))))))))..)))))..)).)).))))).....))))...)))))))))).......))...))

1440 ))))........)))..))).)).)))).....)))).))))((((.....))))..)))))))).))).))((((((......))))..)).))))))((..((.(((..((((.((......))...(((.((......)).)))..))))..)))))

1600 ))).....))++...++...++..++((((..(((((((((((((.....))))...))))))))).....))))..)))).....))).))))((((...((((....((((((((.((..(((......)))...))...))))))))..))))..

1760 ))))))))))).((((.......))))...........(((...((((.(((......))).))))...))))))))).........))))))))))))).))))))....))))).)))))))))))))))..)).)))))).))))(((((...))))

1920 )))))(((..((((((...(((.....))).)))))))))...))))))))......)))))))))...))).))))..)))))))))...)))..((((.......)))).)))))))))((((..((((((...((((.(((((......))))))))

2080 ).))))))...))))....))))))))))))).)))).))))....(((..(((((...........))))))))..)))))))..)))))..

**Table S2. *GQRS mapper* prediction results**

| **Position in *SHR* mRNA** | **Length** | **QGRS** | **G-Score** |
| --- | --- | --- | --- |
| **886** | **30** | **GGCCACGUUUGGACACGUGGCGGCAAACGG** | **19** |
| **1412** | **30** | **GGGUUUGGAGAAUGUUUACGAUGGUUUAGG** | **11** |
| **1587** | **17** | **GGUCGAGGAGGAUGAGG** | **18** |
| **1611** | **16** | **GGUUUGGAGCGGUGGG** | **21** |
| **1668** | **30** | **GGAGAUAUAAAGAAGGUGUUUGGUCGAUGG** | **14** |
| **1747** | **25** | **GGUGGUUUGGGCUAGUGCGUGGCGG** | **13** |

Shaded region highlight the GQ predicted by *ViennaRNA* web server,^[3]^ which shows a high confident prediction in *QGRS mapper*.^[4]^ G score, the likelihood to form a stable G-quadruplex

**Table S3. DNA/RNA sequences used in this study.**

| **Sequence name** | **Sequence** | **Experiments** |
| --- | --- | --- |
| *SHR*-EXON-1 | TTTTCTTGTGTTTCTCGGTG | smFISH probes |
| *SHR*-EXON-2 | CAGCATCATGTGTTGTCTTG | smFISH probes |
| *SHR*-EXON-3 | AAGGGAGACCCACAATATCT | smFISH probes |
| *SHR*-EXON-4 | CGGATTGTTGTTGTTGTTGG | smFISH probes |
| *SHR*-EXON-5 | GGTGGAAGTTCTGCTTAACG | smFISH probes |
| *SHR*-EXON-6 | AAGCATTCTTCGACGACGTC | smFISH probes |
| *SHR*-EXON-7 | AGGAAAGGTCTTCTTCATCC | smFISH probes |
| *SHR*-EXON-8 | TTGTGATGGTTGTGGTGAGA | smFISH probes |
| *SHR*-EXON-9 | TGGGTGGGAGTAGTGAAAGG | smFISH probes |
| *SHR*-EXON-10 | GGAGGAAGGGGTTGATGATG | smFISH probes |
| *SHR*-EXON-11 | GAGTAAGGCGAGGCTAAAGC | smFISH probes |
| *SHR*-EXON-12 | GAACGCGGAAGGGTCATTAT | smFISH probes |
| *SHR*-EXON-13 | CGAAGGACGGAGGAGTTTGA | smFISH probes |
| *SHR*-EXON-14 | CTTCAAGAAGGACCGAGTCT | smFISH probes |
| *SHR*-EXON-15 | AGTGTCTTTGTCGGAGAAGG | smFISH probes |
| *SHR*-EXON-16 | TTGAGCGTCCATAGGATTTG | smFISH probes |
| *SHR*-EXON-17 | TCTCCGTACGGAGAAGAGAG | smFISH probes |
| *SHR*-EXON-18 | GTAAGAAGCCAGTTTTTGCT | smFISH probes |
| *SHR*-EXON-19 | CGGTTGAAGAGAGCTTGGAG | smFISH probes |
| *SHR*-EXON-20 | AAGGAGCAAGTCTTCTCTGT | smFISH probes |
| *SHR*-EXON-21 | TACAGTTTTTCGCGTTGACT | smFISH probes |
| *SHR*-EXON-22 | GGGCTAACTTCTTGGAACTT | smFISH probes |
| *SHR*-EXON-23 | GTCTACTGCTTCCAAGATTG | smFISH probes |
| *SHR*-EXON-24 | ACGTGGAGCTTATGTCAACG | smFISH probes |
| *SHR*-EXON-25 | TAGAAGAGTCGGCCATTGAG | smFISH probes |
| *SHR*-EXON-26 | CTGATCTTGTGGCTAAAGCT | smFISH probes |
| *SHR*-EXON-27 | ACAACTGTGGTTAGCCTTAG | smFISH probes |
| *SHR*-EXON-28 | GCCGTTTGATCGTTGACAAA | smFISH probes |
| *SHR*-EXON-29 | ATCTCTTTCATCATCCGATG | smFISH probes |
| *SHR*-EXON-30 | GAATTTCTCCATTCGGTTTC | smFISH probes |
| *SHR*-EXON-31 | GAAAGGAACTCCCATAAGCC | smFISH probes |
| *SHR*-EXON-32 | CTGGTTTAACGTCGAGTTCG | smFISH probes |
| *SHR*-EXON-33 | TACGCAGTTAATGGCCAAGA | smFISH probes |
| *SHR*-EXON-34 | AACTCGATATCACAGCGTCT | smFISH probes |
| *SHR*-EXON-35 | TCACAATCCTCGGTCTTAAC | smFISH probes |
| *SHR*-EXON-36 | CGACAAGATCAGCTTCTTCT | smFISH probes |
| *SHR*-EXON-37 | AACTCATCATCAAAGCCACC | smFISH probes |
| *SHR*-EXON-38 | AAACATTCTCCAAACCCTCT | smFISH probes |
| *SHR*-EXON-39 | CTCGAAGCAAACCCTAAACC | smFISH probes |
| *SHR*-EXON-40 | CGTCCTTGGAAAACTCTCTT | smFISH probes |
| *SHR*-EXON-41 | CCACAAGATCAACGATCGCA | smFISH probes |
| *SHR*-EXON-42 | CTCCTCAACAAAGCTCTGAC | smFISH probes |
| *SHR*-EXON-43 | ATCGACCAAACACCTTCTTT | smFISH probes |
| *SHR*-EXON-44 | GGCTGATCTCTCCAACAAAG | smFISH probes |
| *SHR*-EXON-45 | AAACAACCCTTTACGTTGGC | smFISH probes |
| *SHR*-EXON-46 | CACACGTGTGAAACCATCTC | smFISH probes |
| *SHR*-EXON-47 | ACTCTCTCAATCTCAAAGCC | smFISH probes |
| *SHR*-EXON-48 | TGTATGAACCTTGATACCCA | smFISH probes |
| *SCR*-EXON-1 | TCTCTCTCGAACTAACCACA | smFISH probes |
| *SCR*-EXON-2 | AATGTTCGTCGTTGTGTGTT | smFISH probes |
| *SCR*-EXON-3 | GGACGAGGAGGACAGTGAAG | smFISH probes |
| *SCR*-EXON-4 | AGGGGTTAGGGGTAATTGAG | smFISH probes |
| *SCR*-EXON-5 | GGATGTGGGTTGGAGATGAA | smFISH probes |
| *SCR*-EXON-6 | CAGTGAGGGGGTGAGAATTT | smFISH probes |
| *SCR*-EXON-7 | ACGATCTCCGATGAGGAGAA | smFISH probes |
| *SCR*-EXON-8 | GATTGAAGGGTTGTTGGTCG | smFISH probes |
| *SCR*-EXON-9 | ACCGGAAGAAGTTGTTCTCA | smFISH probes |
| *SCR*-EXON-10 | TCTTTTTCTCACCATCACTA | smFISH probes |
| *SCR*-EXON-11 | GGGTTAGAAGACATCTCGGA | smFISH probes |
| *SCR*-EXON-12 | GAGGACGAGAGGAGTTGTTG | smFISH probes |
| *SCR*-EXON-13 | AAGAGACGGTGGTTGTTGTG | smFISH probes |
| *SCR*-EXON-14 | TGAGAAGATACAGTAGCCGC | smFISH probes |
| *SCR*-EXON-15 | CCACAAACAGAGAGTGGTGG | smFISH probes |
| *SCR*-EXON-16 | CATTGGTTGTACGGACATCA | smFISH probes |
| *SCR*-EXON-17 | CCATACAGTAGGTGAAGCAG | smFISH probes |
| *SCR*-EXON-18 | AAGGTCTCTGATAATGGCGT | smFISH probes |
| *SCR*-EXON-19 | CCGAGATTTGGGTTACAAGG | smFISH probes |
| *SCR*-EXON-20 | TCGGAGCCTGTATTCAAGAA | smFISH probes |
| *SCR*-EXON-21 | GTTTGAGGAGAAGGGTCAGA | smFISH probes |
| *SCR*-EXON-22 | TGTTGTTGTTGTTGTTGGTG | smFISH probes |
| *SCR*-EXON-23 | CTGAATCGGAGGAGGAGGAG | smFISH probes |
| *SCR*-EXON-24 | GTGGTGCATCGGTAGAAGAA | smFISH probes |
| *SCR*-EXON-25 | TGTAATCCTTCTTCGTCTTG | smFISH probes |
| *SCR*-EXON-26 | CACACTGTAGCAGCAATGTG | smFISH probes |
| *SCR*-EXON-27 | CTTGTTTGCTTCTTCGAGAT | smFISH probes |
| *SCR*-EXON-28 | TCCGAGAAGTAAGCAGCTAC | smFISH probes |
| *SCR*-EXON-29 | ACGAGTTGAGTAATCTCGCT | smFISH probes |
| *SCR*-EXON-30 | TGAAGGCAAAGCCGCGTAAA | smFISH probes |
| *SCR*-EXON-31 | GACCATTTTCAAGCTATGCG | smFISH probes |
| *SCR*-EXON-32 | AGGGCTTATCCCATTAAAGA | smFISH probes |
| *SCR*-EXON-33 | CTGCATGATGTCCAAGTCAA | smFISH probes |
| *SCR*-EXON-34 | TGTGGAATAAACCAGGCCAT | smFISH probes |
| *SCR*-EXON-35 | TTCCATGGAAGTACCAAGTC | smFISH probes |
| *SCR*-EXON-36 | CGAAAGACGTTTCCCTGTAG | smFISH probes |
| *SCR*-EXON-37 | AAAAGGCAGGCCAAGCTTAT | smFISH probes |
| *SCR*-EXON-38 | TTTCTCAGCTAAAGGGCAGA | smFISH probes |
| *SCR*-EXON-39 | AGAGAGTGTGTGCATCAGAG | smFISH probes |
| *SCR*-EXON-40 | TGTTCCACTACTGTCACAAC | smFISH probes |
| *SCR*-EXON-41 | AAGAAAGAACCAGCGTGGCT | smFISH probes |
| *SCR*-EXON-42 | CTCCAACCGCTAATACATTC | smFISH probes |
| *SCR*-EXON-43 | CTCTCAAACTTCACTTCACC | smFISH probes |
| *SCR*-EXON-44 | ACCTTTAAACCCACATTGTT | smFISH probes |
| *SCR*-EXON-45 | TCCGAAGGAAACATTCCCAA | smFISH probes |
| *SCR*-EXON-46 | CCAAGCTTAAGTGTACCATT | smFISH probes |
| *SCR*-EXON-47 | AACTAAGAACGAGGCGTCCA | smFISH probes |
| *SCR*-EXON-48 | ACCAATCAGGTAGCCAATAC | smFISH probes |
| *SHR*-GQ | r(GGUUUGGAGCGGUGGG) | CD |
| *SHR*-GQ_m_ | r(AAUUUGAAGCGAUGAA) | CD |
| *SHR*-GQ_sc_ | r(GGUGUGUGAGCGUGGG) | CD |
| *SHR*-GQ | TGAGCCGTCGGATTCCACGGAGAGGCGAGAGACAGCGAGGAAGTGGTCGAGGAGGATGAGGAATAGTGGGTTTGGAGCGGTGGGGTATAGTGATGAGGTGGCGGATGATGTCAGAGCTTTGTTGAGGAGATATAA | Phase separation |
| *SHR*-GQ_m_ | TGAGCCGTCGGATTCCACGGAGAGGCGAGAGACAGCGAGGAAGTGGTCGAGGAGGATGAGGAATAGTGAATTTGAAGCGATGAAATATAGTGATGAGGTGGCGGATGATGTCAGAGCTTTGTTGAGGAGATATAA | Phase separation |
| *SHR*-GQ_sc_ | TGAGCCGTCGGATTCCACGGAGAGGCGAGAGACAGCGAGGAAGTGGTCGAGGAGGATGAGGAATAGTGGGTGTGTGAGCGTGGGGTATAGTGATGAGGTGGCGGATGATGTCAGAGCTTTGTTGAGGAGATATAA | Phase separation |
| G_2_L_1_ | TGAGCCGTCGGATTCCACGGAGAGGCGAGAGACAGCGAGGAAGTGGTCGAGGAGGATGAGGAATAGTGGTGGTGGTGGTTATAGTGATGAGGTGGCGGATGATGTCAGAGCTTTGTTGAGGAGATATAA | Phase separation |
| G_2_L_2_ | TGAGCCGTCGGATTCCACGGAGAGGCGAGAGACAGCGAGGAAGTGGTCGAGGAGGATGAGGAATAGTGGTTGGTTGGTTGGTTTATAGTGATGAGGTGGCGGATGATGTCAGAGCTTTGTTGAGGAGATATAA | Phase separation |
| G_2_L_3_ | TGAGCCGTCGGATTCCACGGAGAGGCGAGAGACAGCGAGGAAGTGGTCGAGGAGGATGAGGAATAGTGGTTTGGTTTGGTTTGGTTTTATAGTGATGAGGTGGCGGATGATGTCAGAGCTTTGTTGAGGAGATATAA | Phase separation |
| G_2_L_4_ | TGAGCCGTCGGATTCCACGGAGAGGCGAGAGACAGCGAGGAAGTGGTCGAGGAGGATGAGGAATAGTGGTTTTGGTTTTGGTTTTGGTTTTTATAGTGATGAGGTGGCGGATGATGTCAGAGCTTTGTTGAGGAGATATAA | Phase separation |
| G_2_L_5_ | TGAGCCGTCGGATTCCACGGAGAGGCGAGAGACAGCGAGGAAGTGGTCGAGGAGGATGAGGAATAGTGGTTTTTGGTTTTTGGTTTTTGGTTTTTTATAGTGATGAGGTGGCGGATGATGTCAGAGCTTTGTTGAGGAGATATAA | Phase separation |
| G_3_L_1_ | TGAGCCGTCGGATTCCACGGAGAGGCGAGAGACAGCGAGGAAGTGGTCGAGGAGGATGAGGAATAGTGGGTGGGTGGGTGGGTTATAGTGATGAGGTGGCGGATGATGTCAGAGCTTTGTTGAGGAGATATAA | Phase separation |
| G_3_L_2_ | TGAGCCGTCGGATTCCACGGAGAGGCGAGAGACAGCGAGGAAGTGGTCGAGGAGGATGAGGAATAGTGGGTTGGGTTGGGTTGGGTTTATAGTGATGAGGTGGCGGATGATGTCAGAGCTTTGTTGAGGAGATATAA | Phase separation |
| G_3_L_3_ | TGAGCCGTCGGATTCCACGGAGAGGCGAGAGACAGCGAGGAAGTGGTCGAGGAGGATGAGGAATAGTGGGTTTGGGTTTGGGTTTGGGTTTTATAGTGATGAGGTGGCGGATGATGTCAGAGCTTTGTTGAGGAGATATAA | Phase separation |
| G_3_L_4_ | TGAGCCGTCGGATTCCACGGAGAGGCGAGAGACAGCGAGGAAGTGGTCGAGGAGGATGAGGAATAGTGGGTTTTGGGTTTTGGGTTTTGGGTTTTTATAGTGATGAGGTGGCGGATGATGTCAGAGCTTTGTTGAGGAGATATAA | Phase separation |
| G_3_L_5_ | TGAGCCGTCGGATTCCACGGAGAGGCGAGAGACAGCGAGGAAGTGGTCGAGGAGGATGAGGAATAGTGGGTTTTTGGGTTTTTGGGTTTTTGGGTTTTTTATAGTGATGAGGTGGCGGATGATGTCAGAGCTTTGTTGAGGAGATATAA | Phase separation |
| *SHRscramble1-GQ* | GCGTGGCGAGGAAGACGTAGTGAGGAAGGAGCTGTGGAAGAATGAGTAGACCGACGGTGACGCGTCGAGGTTTGGAGCGGTGGGTTGAGAAGTAGTGTAATGTGAGACAACTAGGTATGTTGTGAGGTGCGTGAG | Phase separation |
| *SHRscramble2-GQ* | GATGGCGACGCGGCAGTGAGTGAGCAGAGAGAGAGAGACCTAGCGTGCATGTGTGGAGACGAGAGATGGGTTTGGAGCGGTGGGGTAGGAGTAGCTGATGGTGTTTTAACATGGAGACGTTGGTAGAAATGGAGT | Phase separation |


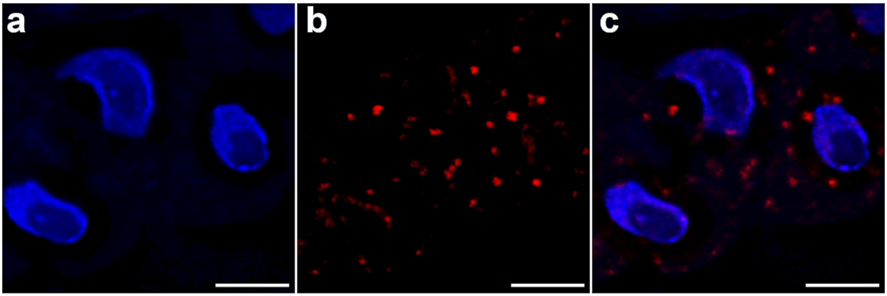


**Figure S1.** *SHR* RNA foci in the root cells of the inducing *SHR* transgenic lines in the *shr-2* mutant. Images generated by smFISH. (a) Nuclei stained with DAPI (blue), *SHR* RNA (red) in (b). Merged image in (c). Scale bar, 10 μm.


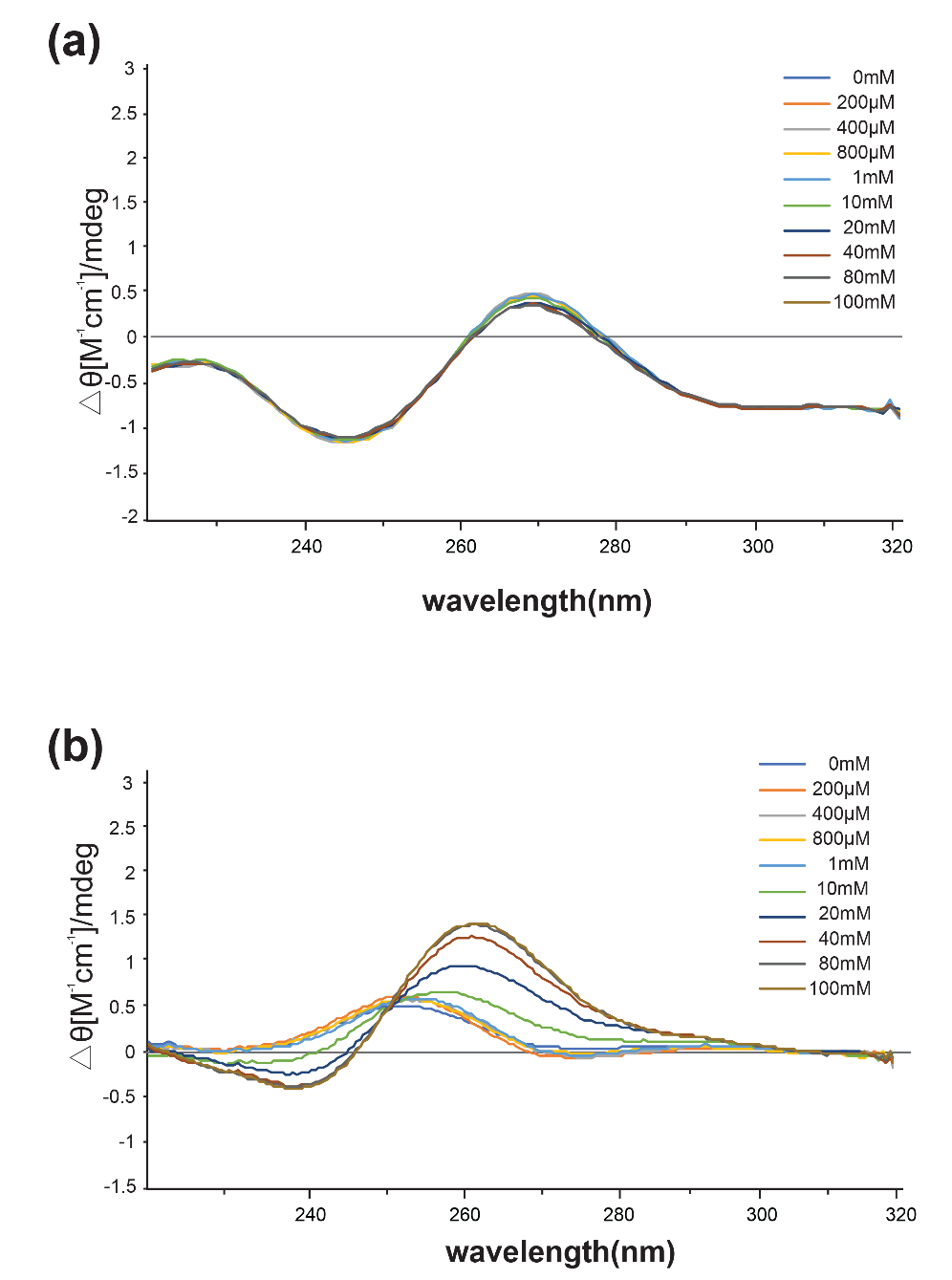


**Figure S2. Circular Dichroism profiles of RNAs.**

CD profiles of *SHR*-GQ_m_ (a) and *SHR*-GQ_sc_ (b) RNAs with potassium ion titration. K^+^ ion induced GQ folding at 10μM RNA in a background of 10mM (pH7.0) lithium cacodylate (LiCac), each trace represents a K^+^ concentration with an average of 4 measurements. K^+^ concentration is increased up to 100mM. The positive peak at ∼260 nm and negative peak at ∼240 nm indicates a parallel topology for the GQ.

**
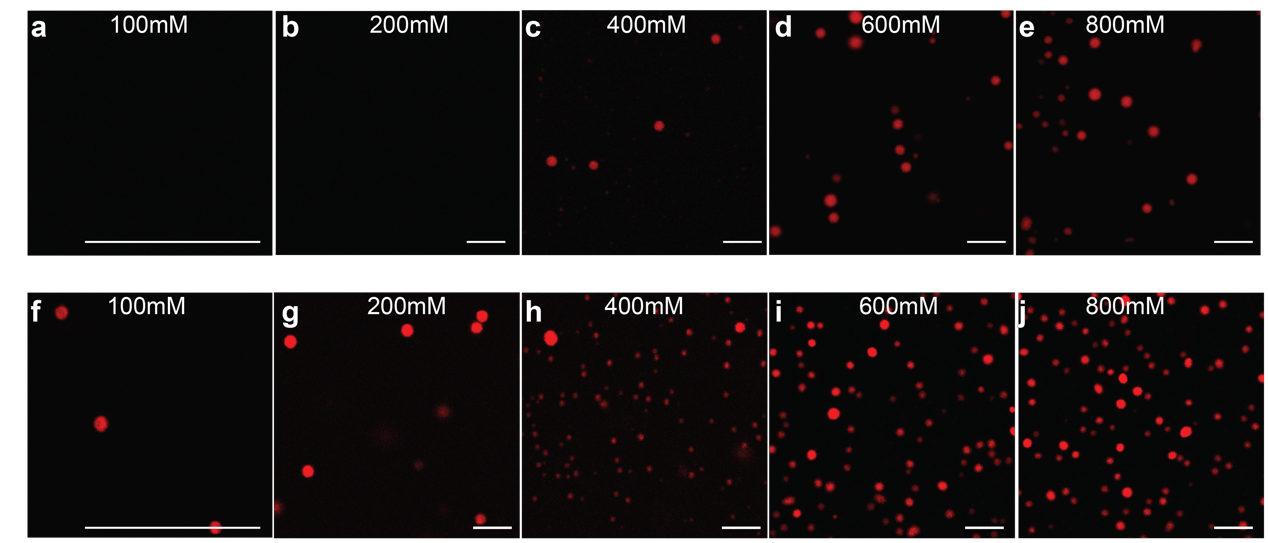
**

**Figure S3. Phase separation of *SHR*-GQ under different conditions.**

a-e. Droplets formed by 0.5 μg/μL *SHR*-GQ RNA with different K^+^ concentrations. f-j. Droplets formed by 1 μg/μL *SHR*-GQ RNA with different K^+^ concentrations. Scale bar=10 μm.


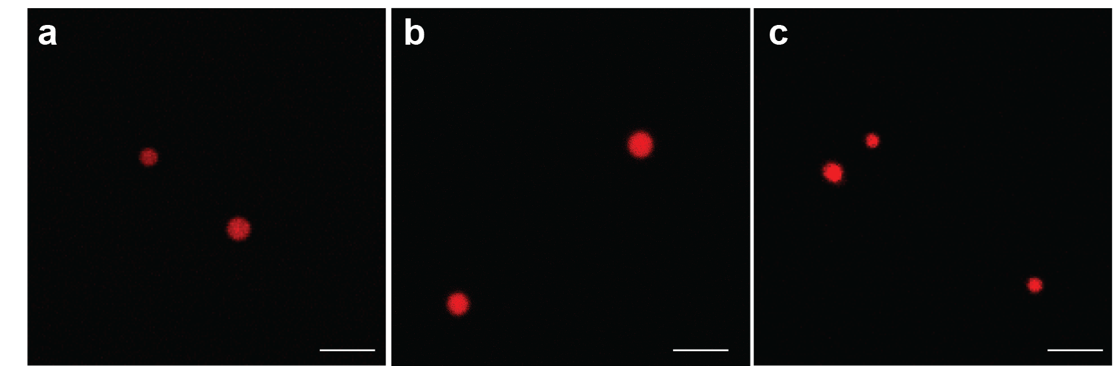


**Figure S4. Droplets formed by *SHR-*GQ (a), *SHRscramble1-*GQ (b) and *SHRscramble2-*GQ (c).**

Fluorescence micrographs of *SHR-*GQ (a) and two RNA, *SHRscramble1-*GQ (b) and *SHRscramble2-*GQ (c) in which the flanking sequences of GQ were scrambled. Experiments were carried out with the RNA concentration of 0.5µg/µL and the K^+^ concentration of 200mM. Scale bars, 5 µm


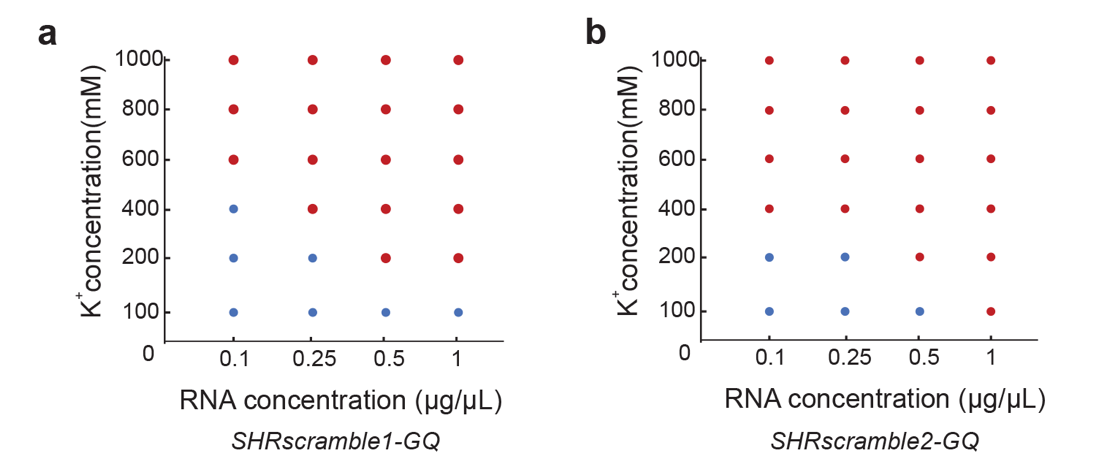


**Figure S5.** **GQ as a general trigger for phase separation.**

Phase diagrams of *SHRscramble1-*GQ (a) and *SHRscramble2-*GQ (b), in which the flanking sequences of GQ were scrambled. Experiments were carried out under varying RNA concentrations and K^+^ concentrations showed in the diagram. Red dots indicate where liquid-liquid-phase-separation happens; Blue dots indicate where liquid-liquid-phase-separation does not occur.


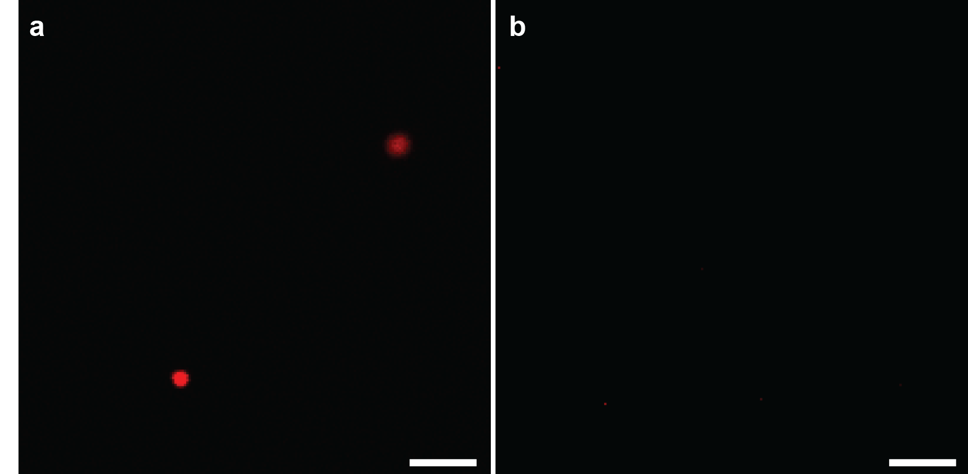


**Figure S6. Full-length *SHR* RNA formed phase separation under physiological conditions.**

Fluorescence micrographs of Full-length *SHR* RNA (a) and full-length *SHR* RNA with GQ mutation (b) at the physiological K^+^ concentration (200mM K^+^). Scale bars, 5 µm

**
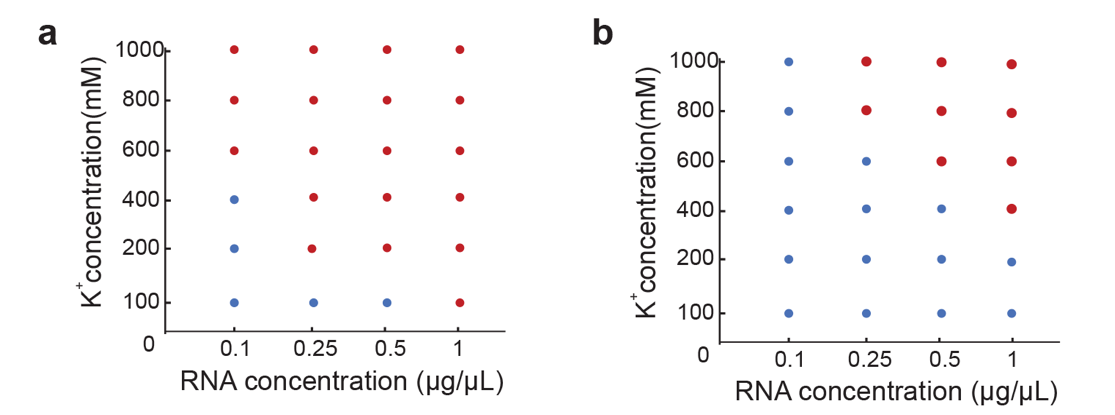
**

**Figure S7. Phase diagram of Full-length *SHR* RNA and Full-length *SHR* RNA with GQ mutation.**

Phase diagrams of Full-length *SHR* RNA (a) and full-length *SHR* RNA with GQ mutation (b). Experiments were carried out under varying RNA concentrations and K^+^ concentrations showed in the diagram. Red dots indicate where liquid-liquid-phase-separation happens; Blue dots indicate where liquid-liquid-phase-separation does not occur.

**
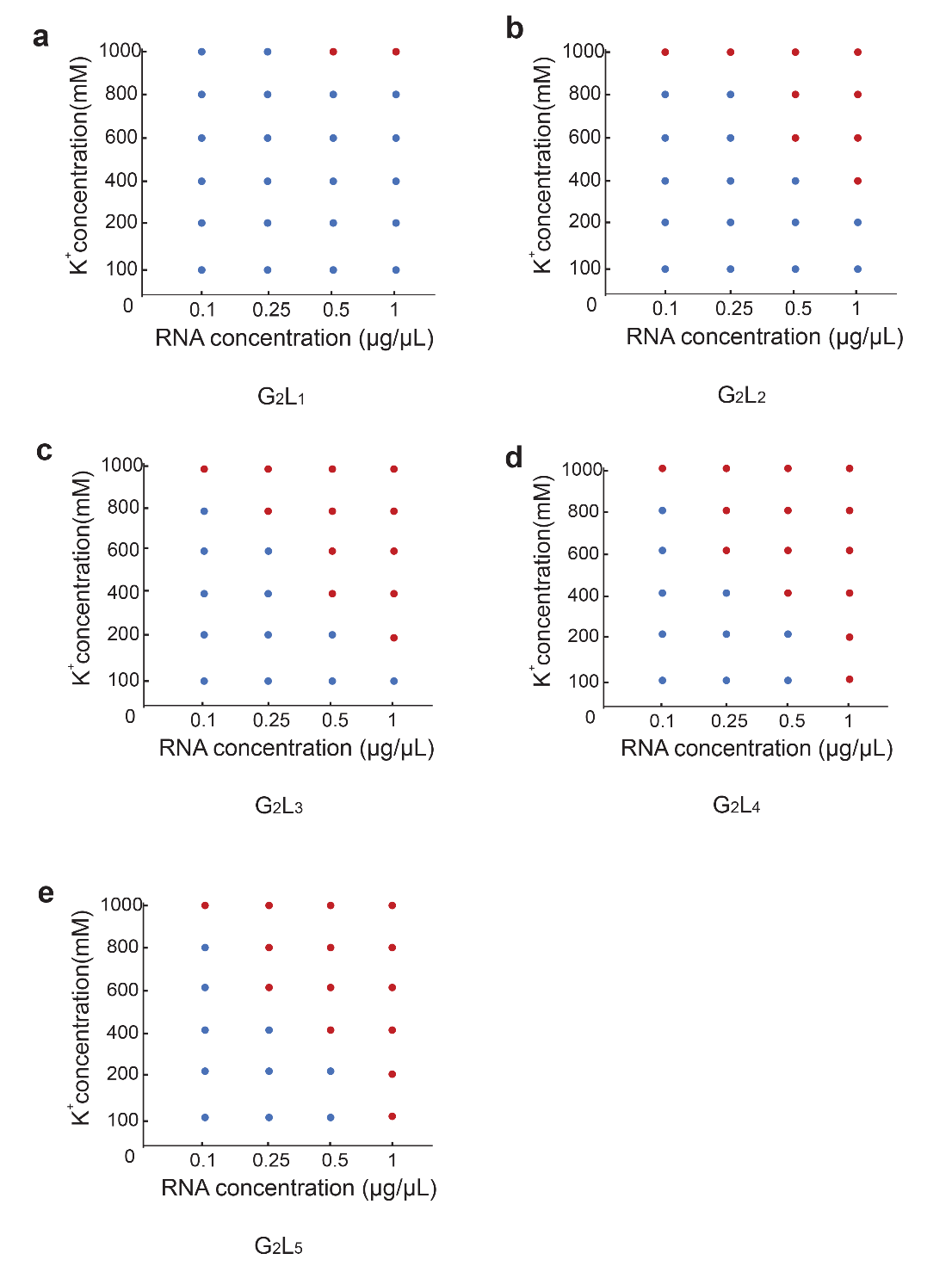
**

**Figure S8. Phase diagram of G2-GQ Forming Sequences.**

Phase diagrams of G_2_L_1_-G_2_L_5_ (a-e) under varying RNA concentrations and K^+^ concentrations. Red dots indicate where liquid-liquid-phase-separation happens; Blue dots indicate where liquid-liquid-phase-separation does not occur.

**
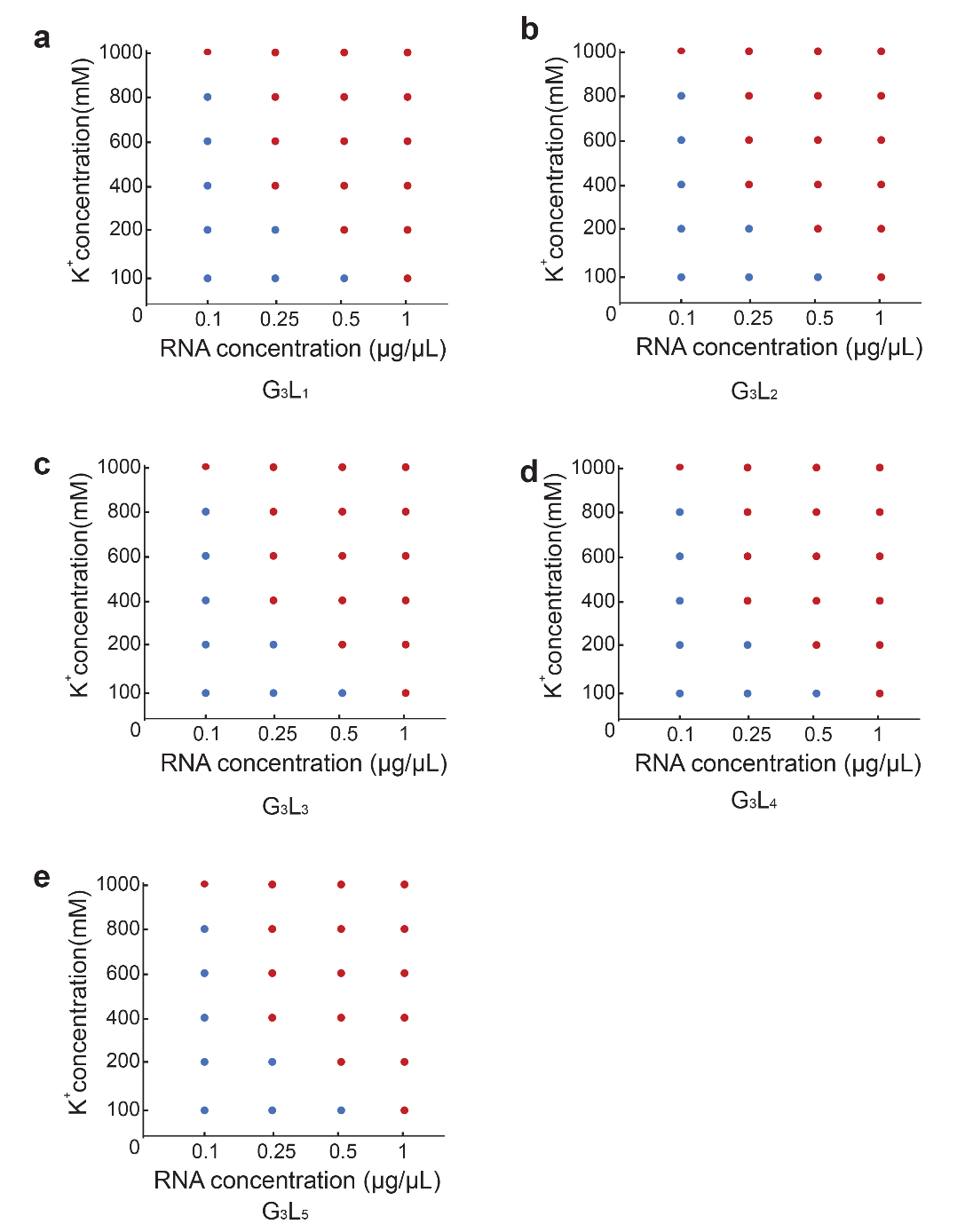
**

**Figure S9. Phase diagram of G3-GQ Forming Sequences.**

Phase diagrams of G_3_L_1_-G_3_L_5_ (a-e) under varying RNA concentrations and K^+^ concentrations. Red dots indicate where liquid-liquid-phase-separation happens; Blue dots indicate where liquid-liquid-phase-separation does not occur.

**Movie S1. Z sections of plant roots with smFISH staining of *SHR* and *SCR* RNAs.**

Raw z stack images demonstrate the smFISH staining of *SHR* (red), *SCR* (green) and DAPI (blue) in plant root cells. The movie shows a merge of the distribution pattern of *SHR* and *SCR* RNAs in 26 z sections. Scale bar, 5 µm

**Movie S2. Z sections of plant roots with smFISH staining of *SHR* in the *SHR* inducing transgenic line.**

Raw z stack images demonstrate the smFISH staining of *SHR* (red), and DAPI (blue) in plant root cells of *SHR* inducing transgenic line. The movie shows a merge of the distribution pattern of *SHR* in 12 z sections. Scale bar, 10 µm

**Movie S3. The quick rearrangement of GQ-triggered droplets**

A time-lapse movie of droplets formed by *SHR*-GQ undergo fusion event. Scale bar,2 µm.

**References**

[1] S. Duncan, T. S. G. Olsson, M. Hartley, C. Dean, S. Rosa, *Bio-protocol* **2017**, *7*, e2240.

[2] C. K. Kwok, S. Balasubramanian, *Angewandte Chemie International Edition* **2015**, *54*, 6751-6754.

[3] I. L. Hofacker, P. F. Stadler, *Bioinformatics* **2006**, *22*, 1172-1176.

[4] O. Kikin, L. D'Antonio, P. S. Bagga, *Nucleic Acids Research* **2006**, *34*, W676-W682.
